# The State of Health in Older Adults in Japan: Trends in Disability, Chronic Medical Conditions and Mortality

**DOI:** 10.1371/journal.pone.0139639

**Published:** 2015-10-02

**Authors:** Shinya Ishii, Sumito Ogawa, Masahiro Akishita

**Affiliations:** Department of Geriatric Medicine, Graduate School of Medicine, The University of Tokyo, Tokyo, Japan; Nagoya University, JAPAN

## Abstract

Both life expectancy and healthy life expectancy in Japan have been increasing and are among the highest in the world, but the gap between them has also been widening. To examine the recent trends in old age disability, chronic medical conditions and mortality in Japan, we retrospectively analyzed three nationally representative datasets: Comprehensive Survey of Living Conditions (2001–2013), Patient Survey (1996–2011) and Vital Statistics (1995–2010). We obtained the sex- and age-stratified trends in disability rate, treatment rates of nine selected chronic medical conditions (cerebrovascular diseases, joint disorders, fractures, osteoporosis, ischemic heart disease, diabetes mellitus, hypertension, pneumonia and malignant neoplasms), total mortality rate and mortality rates from specific causes (cerebrovascular diseases, heart diseases, pneumonia and malignant neoplasms) in both sexes in four age strata (65–69, 70–74, 75–79, 80–84 years). Disability rates declined significantly in both sexes. Treatment rates of all selected medical conditions also decreased significantly, except for fractures in women and pneumonia. Both total mortality rate and cause-specific mortality rates decreased in both sexes. We concluded that the recent decline in disability rates, treatment rates of chronic medical conditions and mortality rates points toward overall improvement in health conditions in adults over the age of 65 years in Japan. Nonetheless, considering the increase in the number of older adults, the absolute number of older adults with disability or chronic medical conditions will continue to increase and challenge medical and long-term care systems.

## Introduction

Japan’s life expectancy has increased steadily over the past several decades and is one of the highest in the world [1–3]. Despite the gradual and continuous rise in life expectancy, the definition of old age has not changed and the age of 65 has been considered the beginning of old age [3, 4]. Consequently, the increase in life expectancy, coupled with the falling birth rate, has resulted in a dramatic increase in the proportion of the “old age” population, from 14.6% in 1995 to 25.1% in 2013 [3]. The shift in demography to older ages has had a large impact on society and the economy, which has led to discussion of the definition of old age [5], since the timing of old age is roughly equivalent to the age of retirement and receiving pension benefit.

The Survey on the Senior Citizens’ Attitude toward Daily Life, conducted by the Cabinet Office of the Government of Japan in 2009, reported that the majority of respondents considered that the threshold for old age should be higher than the current one, 65 years, and more than 40% thought it should be 70 years [6]. This survey also showed that more than a quarter of the respondents answered that an even higher threshold of 75 years is appropriate for old age [6]. This may reflect the change in people’s perception towards aging, and it is possible that the biological age, which involves multitudinous factors including not only elapsed time but also nutrition, living environment and medical conditions, may be going down compared with the chronological age. This hypothesis is compatible with the increase in healthy life expectancy that has occurred simultaneously with the increase in life expectancy [1, 3, 7].

Although both life expectancy and healthy life expectancy have been increasing, the gap between them has also been widening [1, 3]. In order to prevent disability and extend healthy life expectancy, a number of epidemiological studies have been conducted, and comorbid chronic medical conditions have been identified as significant risk factors for disability or the requirement for long-term care [8–13]. However, the prevalence of disability does not necessarily change in parallel with the prevalence of chronic medical conditions. Indeed, studies in some developed countries have reported an increase in the prevalence of chronic medical conditions but a stable or declining disability rate [14–17]. Therefore, examination of the trends in disability and chronic medical conditions in Japan, whose proportion of the old age population is the highest in the world, may facilitate our understanding of the relationship between chronic medical conditions and disability, and the medical and nursing care needs in the old adult population.

In this study, we aimed to characterize the trends in disability and chronic medical conditions over the age of 65 years, the current threshold for old age. We hypothesized that the prevalence of disability, along with the prevalence of chronic medical conditions, has been declining over time, consistent with increasing healthy life expectancy.

## Materials and Methods

All the data analyzed in the current study are publicly available on the Japanese government’s official website, and therefore ethical review is deemed not to be required [18].

We retrospectively analyzed three databases, Comprehensive Survey of Living Conditions, Patient Survey, and Vital Statistics, all of which are conducted by the Ministry of Health, Labour and Welfare and whose details and tabulated data are available on the website of the Statistics Bureau, Ministry of Internal Affairs and Communications [2, 19, 20].

The Comprehensive Survey of Living Conditions is a series of cross-sectional national surveys on a random stratified sample of households and their members [20]. A long-term care questionnaire has been administered every three years starting from 2001, covering persons requiring long-term care (approximately 6000 persons) in 2,500 districts from the National Census. Results from the long-term care questionnaire include the rate of persons certified for long-term care under the Long-Term Care Insurance System per 100,000 population (hereafter referred to as the disability rate), which is approximately equivalent to the prevalence of disability.

The Patient Survey is a series of cross-sectional national surveys on a random sample of medical institutions (including hospitals and outpatient clinics) [19]. All hospitals with more than 500 beds were included in the surveys. Data from medical institutions in Fukushima prefecture and the Ishinomaki and Kesennnuma medical areas of Miyagi prefecture were not included in the 2011 survey data due to the Tohoku earthquake and tsunami on March 11^th^, 2011. The surveys were conducted on one designated date set for each medical institution from three days in October. Physicians filled out the questionnaire and collected information on patients who attended the participating medical institutions on the date of the survey. Approximately 8 million patients were included in each survey. The International Statistical Classification of Diseases and Related Health Problems, ninth Revision (ICD–9), published by the World Health Organization, was applied to classify diseases and injuries in the surveys until 1996, and the ICD–10 thereafter, which prevented direct comparison of the data prior to and after 1996.

For each disease or injury, the rate of estimated number of patients per 100,000 population (hereafter referred to as the treatment rate) was calculated as the estimated number of patients divided by the estimated population x 100,000. The estimated number of patients who continuously received medical care was calculated using the following formula [19]:

Estimated number of patients receiving medical treatment = Estimated number of inpatients + Estimated number of initial visit outpatients + (Estimated number of return visit outpatients × Average interval since last visit × Adjustment factor (6/7))

Therefore, the estimated number of patients included those who did not receive medical care at medical institutions on the date of the survey, and the treatment rate can be considered a rough approximation of the prevalence.

Our analysis focused on the trends in the prevalence of medical conditions that can cause disability and lower healthy life expectancy. The Comprehensive Survey of Living Conditions in 2013 reported that the most common cause of disability was cerebrovascular accident, followed by dementia, frailty due to aging, joint disorders, bone fracture and cardiac disease [20]. These six categories accounted for more than 70% of causes of disability. In the current analysis, we chose to investigate cerebrovascular diseases, osteoarthritis, inflammatory polyarthropathies, fractures, osteoporosis, ischemic heart disease, diabetes mellitus, hypertension, pneumonia and malignant neoplasms based on their clinical significance, their potential to cause disability, and the availability of data. Osteoarthritis and inflammatory polyarthropathies were combined into a single category, joint disorders. We initially planned to investigate Alzheimer’s disease as well, but our preliminary analysis suggested that the treatment rate had been much lower than the prevalence previously reported [21] despite recent increase in the treatment rate, presumably because of under-diagnosis (S1 Fig and S1 Table) [22]. We therefore decided not to include Alzheimer’s disease in our analysis because our primary aim was to examine the actual prevalence of chronic medical conditions.

Vital Statistics is based on the Family Registry, and collects data on birth, marriage and death registrations [2]. The data on total mortality rate and mortality rates from specific causes (cerebrovascular diseases, heart diseases, pneumonia and malignant neoplasms) were obtained from Vital Statistics. The cause of death was obtained from the death certificate issued by physicians, and classified using ICD–9 until 1995, and ICD–10 thereafter, and therefore we included the data after 1995 in our analysis.

### Statistical Analysis

We stratified the data by sex and examined the trends in disability rate, treatment rates of selected medical conditions and mortality rates in the four 5-year age strata over the age of 65 years (65–69, 70–74, 75–79 and 80–84 years). The overall increasing or decreasing trend in the rates in each sex was evaluated by the linear trend test using PROC GLM of SAS (SAS Institute, Inc., Cary, NC, USA) adjusted for the age groups. Provided the initial trend test was statistically significant, linear regression was performed to evaluate the trend in each age stratum. Two-sided p<0.05 was considered statistically significant.

## Results

### Trends in Disability

**Fig 1:**
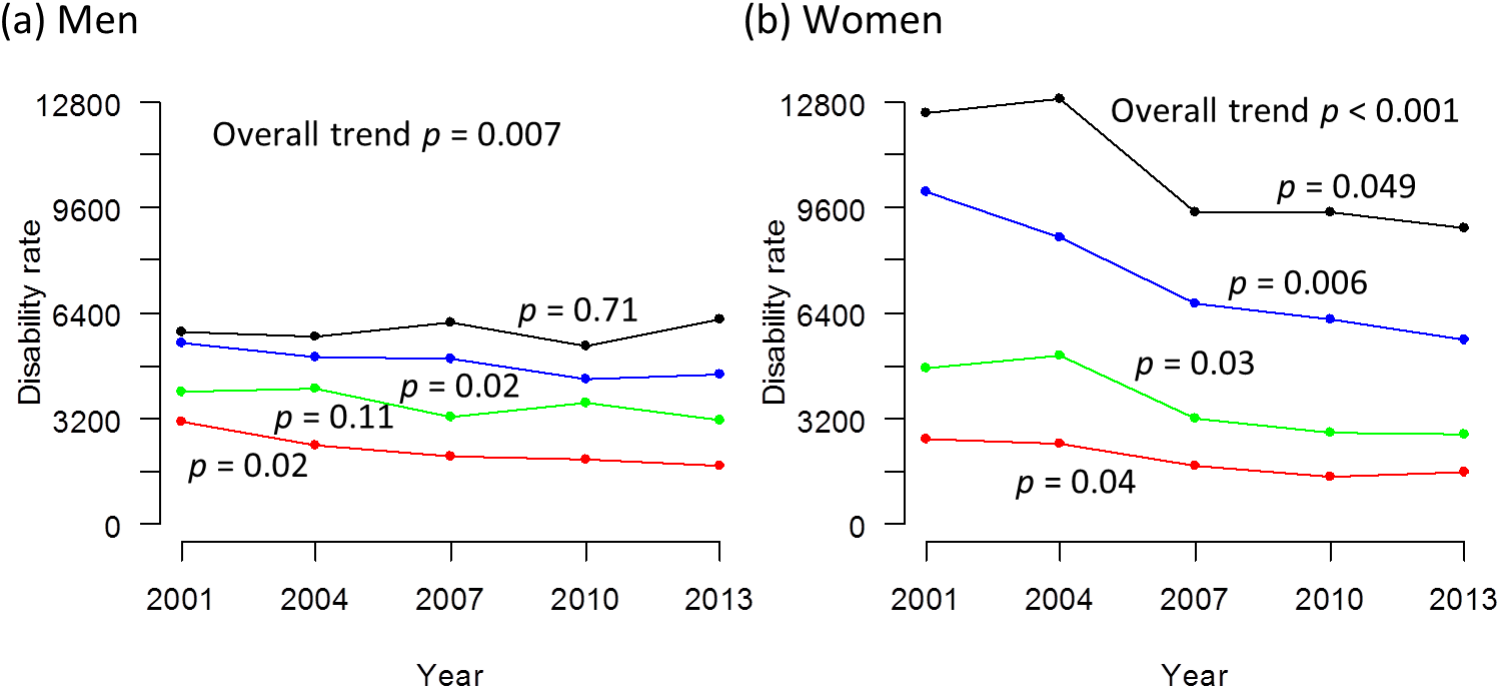
Trends in disability rate in men and women from 2001 to 2013. (a) men. (b) women. The disability rate is the rate of persons certified for long-term care under the Long-Term Care Insurance System per 100,000 population. The black line represents those aged 80–84 years, the blue line represents those aged 75–79 years, the green line represents those aged 70–74 years and the red line represents those aged 65–69 years. The p values signify statistical significance for the trends in each age stratum.

The trends in the disability rates from 2001 to 2013 are displayed in Fig 1 (Tabulated data available in S2 Table). The overall trend was downward and statistically significant in each sex. The trend in each age stratum was also statistically significant except for men aged 70–74 and 80–84 years.

### Trends in Chronic Medical Conditions

**Fig 2:**
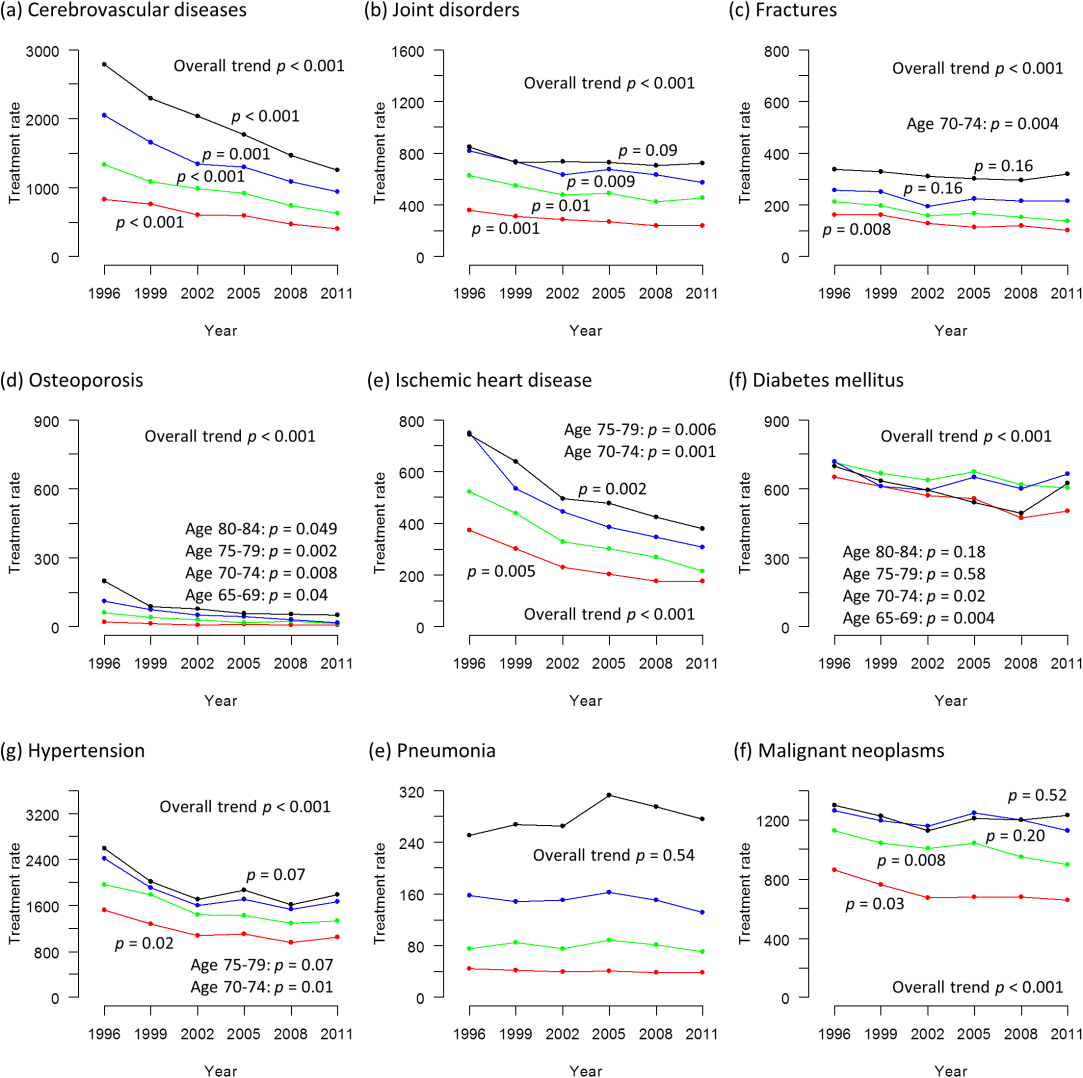
Trends in treatment rates of nine selected medical conditions in men from 1996 to 2011. (a) cerebrovascular diseases (b) joint disorders (c) fractures (d) osteoporosis (e) ischemic heart disease (f) diabetes mellitus (g) hypertension (h) pneumonia (i) malignant neoplasms The treatment rate is calculated as the estimated number of patients divided by the estimated population x 100,000. The black line represents those aged 80–84 years, the blue line represents those aged 75–79 years, the green line represents those aged 70–74 years and the red line represents those aged 65–69 years. The p values signify statistical significance for the trends in each age stratum.

**Fig 3:**
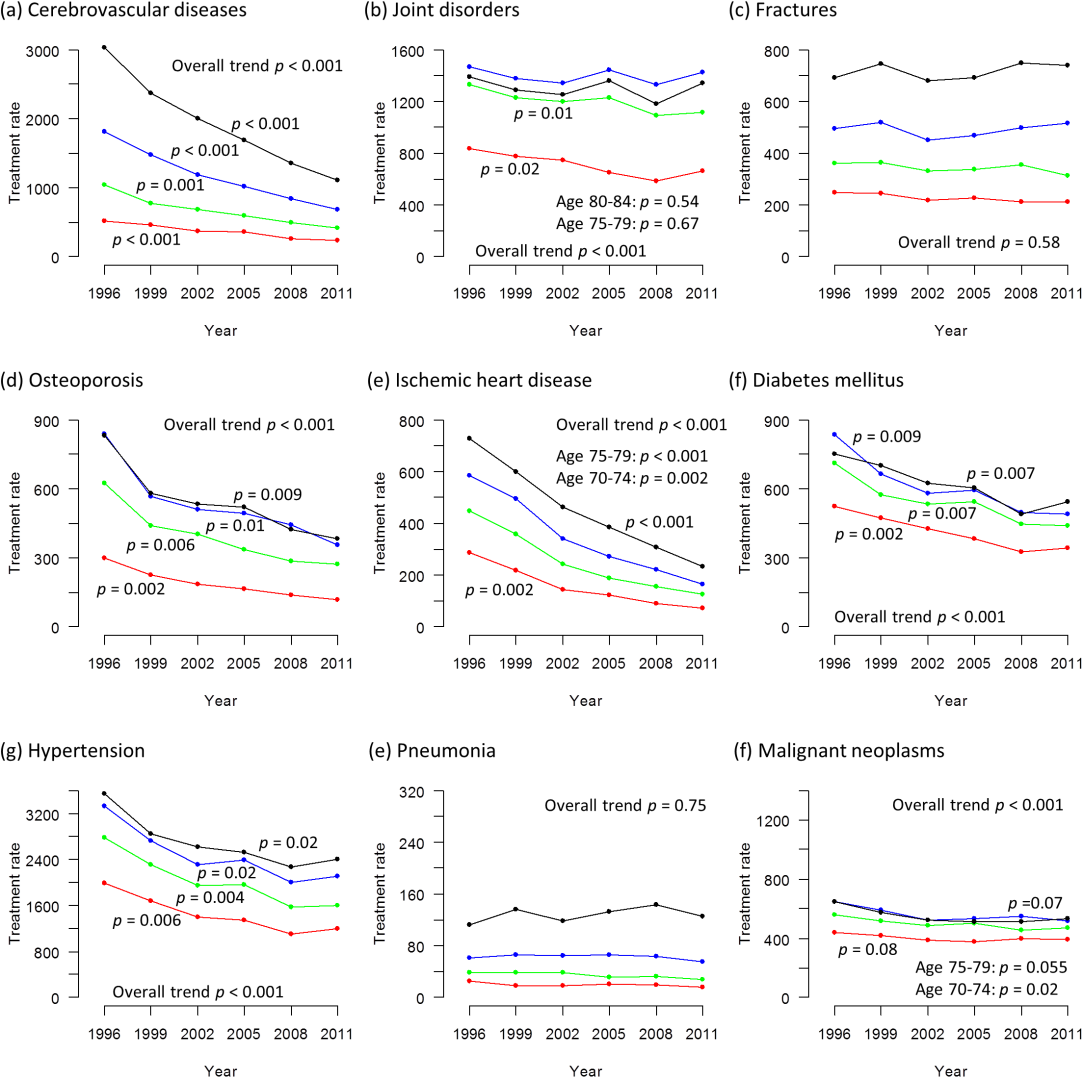
Trends in treatment rates of nine selected medical conditions in women from 1996 to 2011. (a) cerebrovascular diseases (b) joint disorders (c) fractures (d) osteoporosis (e) ischemic heart disease (f) diabetes mellitus (g) hypertension (h) pneumonia (i) malignant neoplasms The treatment rate is calculated as the estimated number of patients divided by the estimated population x 100,000. The black line represents those aged 80–84 years, the blue line represents those aged 75–79 years, the green line represents those aged 70–74 years and the red line represents those aged 65–69 years. The p values signify statistical significance for the trends in each age stratum.

Figs 2 and 3 show the trends in the treatment rates for the nine selected medical conditions from 1996 to 2011 (Tabulated data available in S3 Table). The overall treatment rate declined significantly for all medical conditions in each sex, except for fractures in women and pneumonia.

For cerebrovascular diseases, ischemic heart disease and osteoporosis, the downward trend was statistically significant for all age strata (65–69, 70–74, 75–79 and 80–84) in each sex.

For diabetes mellitus and hypertension, the treatment rate decreased over time with statistical significance for all age strata in women, but in men the downward trend was statistically significant in two younger age strata only (65–69 and 70–74).

For fractures and malignant neoplasms, the downward trend was statistically significant in two younger age strata of 65–69 and 70–74 years in men. In women, statistical significance was observed only in the age stratum of 70–74 years for malignant neoplasms.

For joint disorders, the downward trend was statistically significant in two younger age strata of 65–69 and 70–74 years in each sex, but the significance decline was not observed in older age strata except for the age stratum of 75–79 years in men.

### Trends in Mortality

**Fig 4:**
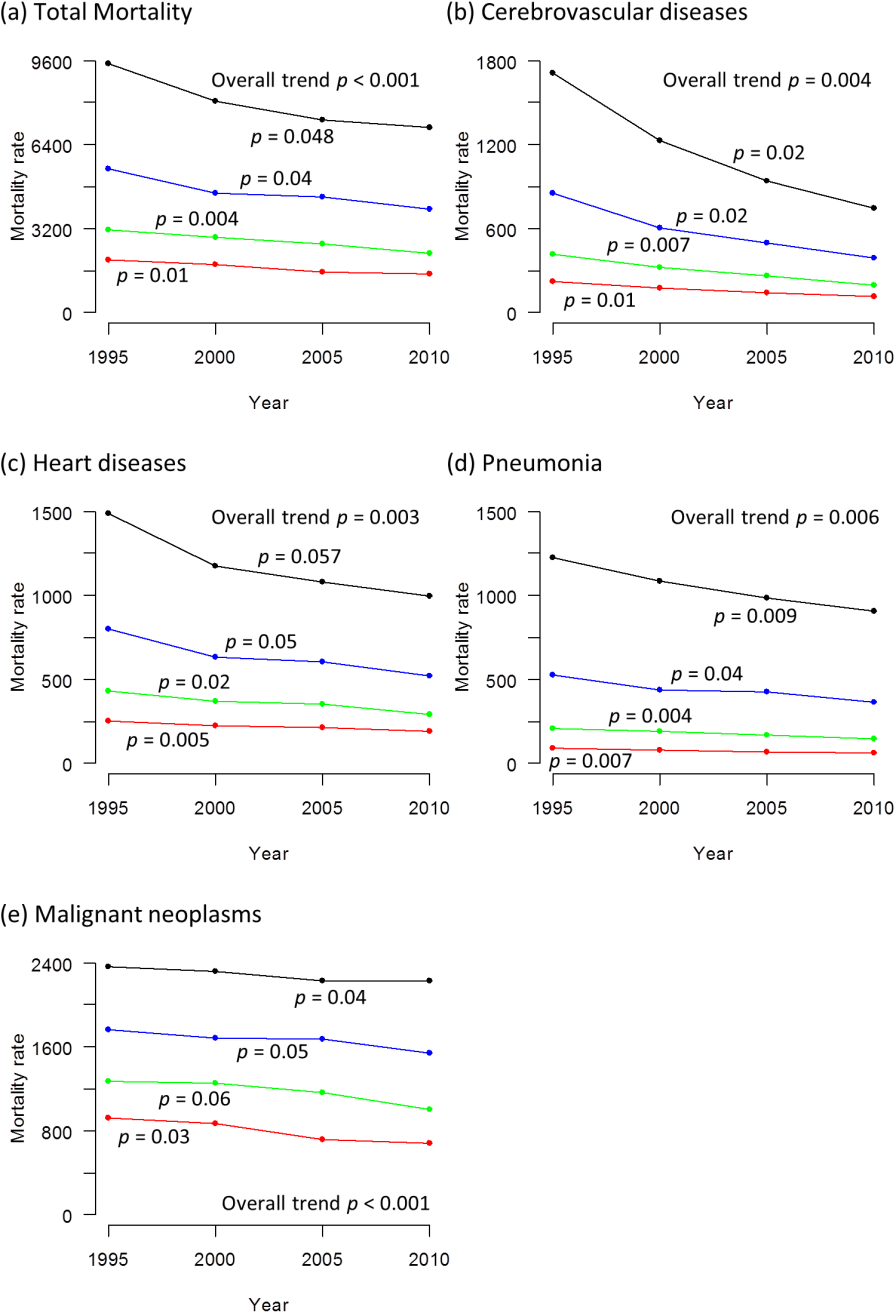
Trends in total mortality rate and mortality rates from specific causes in men from 1995 to 2010. (a) total mortality rate. Mortality rate from (b) cerebrovascular diseases (c) heart diseases (d) pneumonia (e) malignant neoplasms. The mortality rate is calculated as the number of deceased divided by the estimated population x 100,000. The black line represents those aged 80–84 years, the blue line represents those aged 75–79 years, the green line represents those aged 70–74 years and the red line represents those aged 65–69 years. The p values signify statistical significance for the trends in each age stratum.

**Fig 5:**
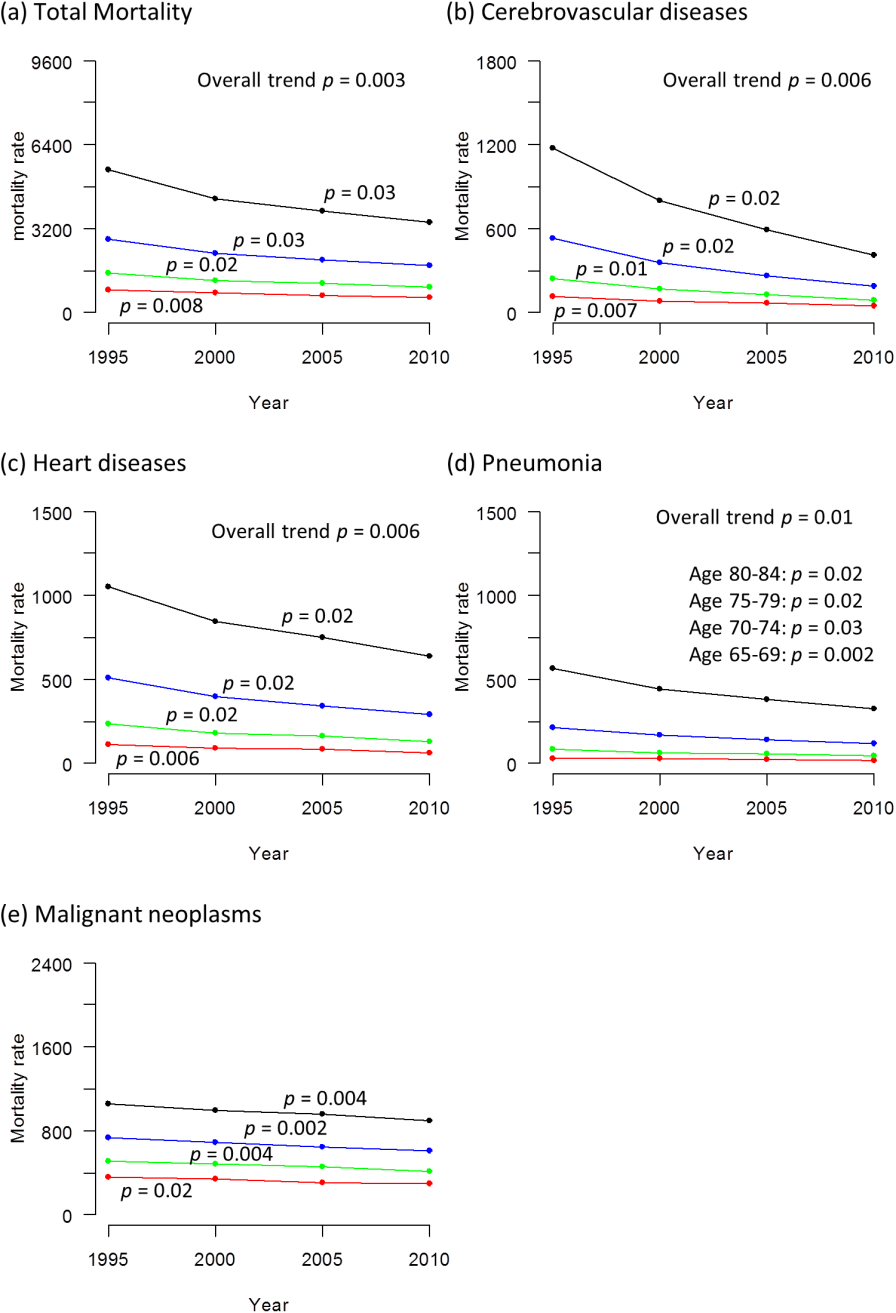
Trends in total mortality rate and mortality rates from specific causes in women from 1995 to 2010. (a) total mortality rate. Mortality rate from (b) cerebrovascular diseases (c) heart diseases (d) pneumonia (e) malignant neoplasms. The mortality rate is calculated as the number of deceased divided by the estimated population x 100,000. The black line represents those aged 80–84 years, the blue line represents those aged 75–79 years, the green line represents those aged 70–74 years and the red line represents those aged 65–69 years. The p values signify statistical significance for the trends in each age stratum.

Figs 4 and 5 show the trends in total mortality rate and mortality rates from specific causes from 1995 to 2010 (Tabulated data available in S4 Table). Both total mortality rate and cause-specific mortality rates declined significantly in all age strata in both sexes, except for mortality rate from malignant neoplasms in the age stratum of 70–74 years and heart diseases in the age stratum of 80–84 years in men in which the change was not statistically significant.

## Discussion

In the analysis of nationally representative datasets, we demonstrated that the disability rate and mortality rate declined significantly over time in old adults aged between 65 and 84 in Japan. We also observed a decrease in treatment rates of many chronic medical conditions over approximately the same time period. This finding suggests a decline in the prevalence of these conditions, which is in concordance with the decline in disability rate. The concurrent decrease in mortality implies that the decrease in treatment rates is not attributable to attrition of sicker persons or survival bias. Hence, combined together, our findings indicate overall improvement in health conditions among adults entering “old age”; that is, people reaching the age of 65 years have recently enjoyed better health compared to preceding cohorts.

Among conditions whose treatment rates declined, three medical conditions, namely cerebrovascular diseases, ischemic heart disease and osteoporosis, showed a particularly substantial decline. The treatment rates of these medical conditions in each age group in 2011 were roughly equivalent to or even lower than the treatment rates of the group 5 years younger in 1996, and were consistent in each sex. Public health organizations have made efforts to increase people’s awareness of the importance of a healthy lifestyle and avoiding major risk factors for these medical conditions, such as smoking, hypertension and diabetes. These efforts have gradually altered people’s behavior. The smoking rate was 44.7% for men over 60 and 7.8% for women over 60 in 1996, but in 2011 it fell to 23.9% and 6.4%, respectively [23]. People, particularly those older than 60, exercise more than previously [23]. The Specific Health Checkup, which focuses on screening for metabolic syndrome and lifestyle-related diseases, was introduced in 2008 against a backdrop of increased awareness of such medical conditions, and led to early screening, diagnosis and treatment [24]. In addition, advances in medical technology, medication and care and an improving standard of living have helped facilitate prevention and management of these conditions. It should be noted that cerebrovascular diseases and ischemic heart disease are important causes of both mortality and disability.

Interestingly, the change in the overall treatment rate for fracture in women was not statistically significant, whereas the treatment rate for osteoporosis declined significantly. The observed disparity between the treatment rates of fractures and osteoporosis may be due to limited predictive ability of bone mineral density measurements, which have been widely used to diagnose osteoporosis. It is true that fracture is more likely to occur when bone mineral density is lower, but multiple factors play a role in determining fracture risk, and bone mineral density alone accounts for only 1.7 to 7.4 percent of fracture risk [25, 26]. Another possible explanation for the disparity may be under-diagnosis of osteoporosis. In primary care settings, osteoporosis is often undiagnosed and untreated. However, health checkups for old adults in Japan include screening for osteoporosis, and it is unlikely that the diagnostic rate of osteoporosis has significantly changed recently. The time gap between osteoporosis and fractures may also help explain the disparity. People tend to suffer fracture at an older age than the age when they receive a diagnosis of osteoporosis. Therefore, even though the treatment rate for osteoporosis has declined, the effects on the treatment rate of fracture may be delayed and take some more years to be observable.

The treatment rates for diabetes mellitus and hypertension declined significantly for all age strata in women, but the improvement in men was mostly restricted to those younger than 75 years. The reason for this apparent disparity between men and women is not clear, but it may reflect sex differences in age-related changes or a cohort effect. Those older than 75 years experienced the Second World War in their childhood when chronic malnutrition was widespread and started their occupational career in the period of rapid growth in 1960s when men were expected to earn their livings and women to stay home and raise a family.

An improvement in the treatment rate of pneumonia was not observed in each sex. Despite the largely unchanged treatment rate of pneumonia, pneumonia-specific mortality rate declined over time. This suggests stable occurrence of pneumonia, but possibly of a less severe form, and improved management of pneumonia. Because effective preventive measures for pneumonia, such as pneumococcal or influenza vaccination, are already widely available for old adults, further improvement in the prevention of pneumonia may be hard to achieve.

Only a few studies have provided descriptive epidemiological data on disability and chronic diseases among older adults in Japan. The Analysis of National Survey of Japanese Elderly, a nationally representative six-wave panel study, reported that six out of ten measures of Activity of Daily Living (ADL) and instrumental ADL improved significantly after adjustment for age from 1993 to 2002 [27]. A study of the Comprehensive Survey of Living Conditions reported that the number of expected years of life without activity limitation increased from 1995 to 2004 [7]. These studies reported data more than 10 years ago, but the results are consistent with our findings, implying that the improvement in health conditions started earlier than our data coverage.

Our study has some limitations that need to be acknowledged. First, the Patient Survey collected information on diseases and injuries from physicians, but the diagnostic criteria were not standardized and their severity was not included in the questionnaire. All medical conditions may not be captured by the questionnaire in the case of patients with multiple comorbidity, which is particularly concerning for older adults because older adults tend to have an increased number of comorbid conditions [28]. Second, the Patient Survey is conducted every three years in October, and therefore seasonal variation in the treatment rate is not accounted for. However, because each survey was conducted in a standardized manner at the same time of the year, the survey at least can provide valid estimates of the trends in the treatment rates over years. Third, some medical conditions may be underdiagnosed and not well captured by the survey. We did not include Alzheimer’s disease in our analysis because it appears to be consistently underdiagnosed, and the change in the treatment rate of Alzheimer’s disease does not seem to reflect the true change in prevalence. Last, the Patient Survey data in 2011 did not include data from medical institutions in Fukushima prefecture and the Ishinomaki and Kesennnuma medical areas of Miyagi prefecture. However, the populations in these areas comprised less than 3% of the population of Japan, and exclusion of data from these areas was unlikely to bias our findings and alter our conclusion.

Despite these limitations, our study has a number of strengths. The databases we utilized are nationally representative, containing large numbers of participants. The Vital Statistics in Japan is highly reliable, and the ascertainment of causes of death was based on death certificates issued by physicians. The data were collected in a standardized manner over a couple of decades, providing a unique opportunity to obtain descriptive epidemiological data of disability, comorbid medical conditions and mortality.

Our findings have several implications. First, even though our findings indicate overall improvement in health conditions in the population aged between 65 and 84, the absolute number of older adults with chronic medical conditions or disability will continue to increase as a result of increase in older adults [3]. This will be a great challenge for the National Health Insurance system and the Long-Term Care Insurance system in Japan. Second, considering the downward trajectory of the treatment rates observed in many medical conditions, it is important to keep raising the general public’s awareness of taking preventive measures to help stave off these conditions. This is a particular priority when we consider the ramifications of the increasing number of older adults with disability. Third, the baby boomers, who were born after the Second World War, are now entering old age. It is important to carefully monitor their health conditions to see if the improvement in health conditions observed in this study will continue. Lastly, our findings do not explain the widening gap between life expectancy and healthy life expectancy. Further research focusing on very old adults is warranted.

We conclude that the prevalence of many chronic medical conditions has declined among old adults over time in Japan. Coupled with the decline in disability rate and mortality rate, our findings may signify overall improvement in health conditions among old adults. This is consistent with improving healthy life expectancy and supports the hypothesis that biological age may be getting lower compared with chronological age. Nonetheless, the increase in number of older adults will offset the improvement in health conditions, and older adults with chronic medical conditions or disability will continue to increase. Therefore, continuous public health efforts to prevent chronic medical conditions and a roadmap for a health care system to meet the increasing health care needs of older adults are still warranted.

## Supporting Information

S1 Fig Trends in treatment rates of Alzheimer’s disease in men and women from 1996 to 2011. The treatment rate is calculated as the estimated number of patients divided by the estimated population x 100,000. The black line represents those aged 80–84 years, the blue line represents those aged 75–79 years, the green line represents those aged 70–74 years and the red line represents those aged 65–69 years. The p values signify statistical significance for the trends in each age stratum.
(TIF)

S1 Table Trends in treatment rates of Alzheimer’s disease in men and women from 1996 to 2011. The treatment rate is calculated as the estimated number of patients divided by the estimated population x 100,000.
(DOCX)

S2 Table Trends in disability rate in men and women from 2001 to 2013. The disability rate is the rate of persons certified for long-term care under the Long-Term Care Insurance System per 100,000 population.
(DOCX)

S3 Table Trends in treatment rates of nine selected medical conditions in men and Women from 1996 to 2011. The treatment rate is calculated as the estimated number of patients divided by the estimated population x 100,000.
(DOCX)

S4 Table Trends in total mortality rate and mortality rates from specific causes in men and women from 1995 to 2010. The mortality rate is calculated as the number of deceased divided by the estimated population x 100,000.
(DOCX)
