# Supplementary figures and images for: The State of Health in Older Adults in Japan: Trends in Disability, Chronic Medical Conditions and Mortality
Source: PLoS One. 2015 Oct 2;10(10):e0139639. doi: 10.1371/journal.pone.0139639 (PMC4592221; doi:10.1371/journal.pone.0139639)

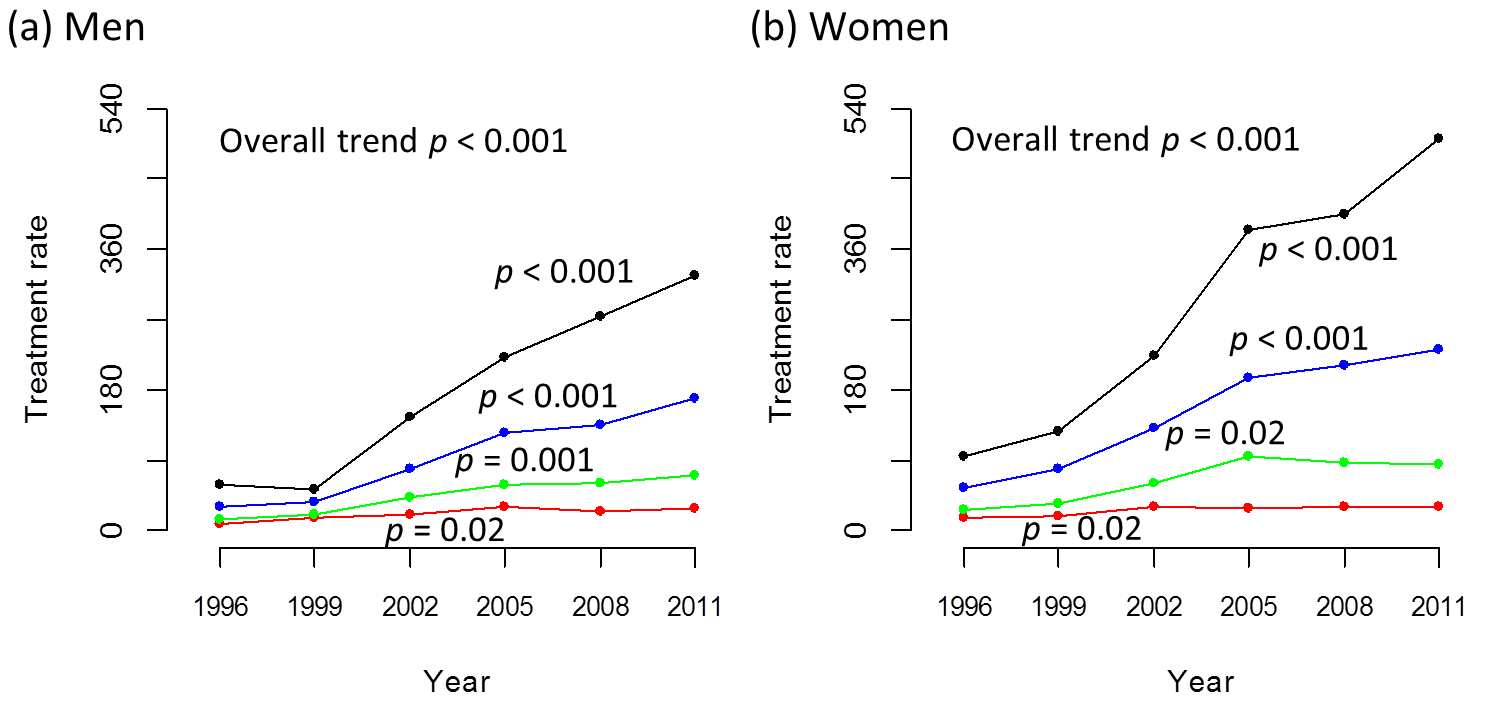

Supplement: S1 Fig — The treatment rate is calculated as the estimated number of patients divided by the estimated population x 100,000. The black line represents those aged 80–84 years, the blue line represents those aged 75–79 years, the green line represents those aged 70–74 years and the red line represents those aged 65–69 years. The p values signify statistical significance for the trends in each age stratum. (TIF) [file pone.0139639.s001.tif]
