# Supplementary material for: The State of Health in Older Adults in Japan: Trends in Disability, Chronic Medical Conditions and Mortality
Source: PLoS One. 2015 Oct 2;10(10):e0139639. doi: 10.1371/journal.pone.0139639 (PMC4592221; doi:10.1371/journal.pone.0139639)
Supplement: S1 Table — The treatment rate is calculated as the estimated number of patients divided by the estimated population x 100,000. (DOCX) [file pone.0139639.s002.docx]

**S1 Table. Trends in treatment rates of Alzheimer’s disease in men and women from 1996 to 2011**

|  |  | Age | Year | | | | | | p for overall trend | p for trend |
| --- | --- | --- | --- | --- | --- | --- | --- | --- | --- | --- |
|  |  |  | 1996 | 1999 | 2002 | 2005 | 2008 | 2011 |  |  |
| Alzheimer’s Disease | Men | 65-69 | 9 | 16 | 20 | 31 | 25 | 29 | <0.001 | 0.02 |
|  |  | 70-74 | 14 | 21 | 43 | 59 | 61 | 71 |  | 0.001 |
|  |  | 75-79 | 30 | 37 | 79 | 125 | 135 | 169 |  | <0.001 |
|  |  | 80-84 | 58 | 53 | 146 | 222 | 275 | 326 |  | <0.001 |
|  | Women | 65-69 | 17 | 18 | 30 | 28 | 31 | 31 | <0.001 | 0.02 |
|  |  | 70-74 | 27 | 34 | 61 | 96 | 88 | 85 |  | 0.02 |
|  |  | 75-79 | 54 | 80 | 132 | 195 | 212 | 232 |  | <0.001 |
|  |  | 80-84 | 96 | 128 | 223 | 385 | 404 | 502 |  | <0.001 |

The treatment rate is calculated as the estimated number of patients divided by the estimated population x 100,000.
