# Supplementary material for: The State of Health in Older Adults in Japan: Trends in Disability, Chronic Medical Conditions and Mortality
Source: PLoS One. 2015 Oct 2;10(10):e0139639. doi: 10.1371/journal.pone.0139639 (PMC4592221; doi:10.1371/journal.pone.0139639)
Supplement: S2 Table — The disability rate is the rate of persons certified for long-term care under the Long-Term Care Insurance System per 100,000 population. (DOCX) [file pone.0139639.s003.docx]

S2 Table. Trends in disability rate in men and women from 2001 to 2013

|  | Age | Year | | | | | p for overall trend | p for trend |
| --- | --- | --- | --- | --- | --- | --- | --- | --- |
|  |  | 2001 | 2004 | 2007 | 2010 | 2013 |  |  |
| Men | 65-69 | 3,129 | 2388 | 2063 | 1993 | 1765 | 0.007 | 0.02 |
|  | 70-74 | 4,030 | 4097 | 3251 | 3703 | 3164 |  | 0.11 |
|  | 75-79 | 5,509 | 5089 | 5039 | 4419 | 4523 |  | 0.02 |
|  | 80-84 | 5,822 | 5686 | 6124 | 5416 | 6189 |  | 0.71 |
| Women | 65-69 | 2,601 | 2450 | 1784 | 1456 | 1586 | <0.001 | 0.03 |
|  | 70-74 | 4,746 | 5123 | 3225 | 2802 | 2728 |  | 0.04 |
|  | 75-79 | 10,070 | 8700 | 6705 | 6205 | 5569 |  | 0.006 |
|  | 80-84 | 12,428 | 12860 | 9439 | 9425 | 8973 |  | 0.049 |

The disability rate is the rate of persons certified for long-term care under the Long-Term Care Insurance System per 100,000 population.
