# Supplementary material for: The State of Health in Older Adults in Japan: Trends in Disability, Chronic Medical Conditions and Mortality
Source: PLoS One. 2015 Oct 2;10(10):e0139639. doi: 10.1371/journal.pone.0139639 (PMC4592221; doi:10.1371/journal.pone.0139639)
Supplement: S3 Table — The treatment rate is calculated as the estimated number of patients divided by the estimated population x 100,000. (DOCX) [file pone.0139639.s004.docx]

S3 Table. Trends in treatment rates of nine selected medical conditions in men and women from 1996 to 2011

|  |  | Age | Year | | | | | | p for overall trend | p for trend |
| --- | --- | --- | --- | --- | --- | --- | --- | --- | --- | --- |
|  |  |  | 1996 | 1999 | 2002 | 2005 | 2008 | 2011 |  |  |
| Cerebrovascular diseases | Men | 65-69 | 834 | 758 | 609 | 598 | 468 | 410 | <0.001 | <0.001 |
|  |  | 70-74 | 1334 | 1085 | 991 | 919 | 742 | 626 |  | <0.001 |
|  |  | 75-79 | 2052 | 1657 | 1349 | 1299 | 1088 | 940 |  | 0.001 |
|  |  | 80-84 | 2780 | 2288 | 2032 | 1764 | 1469 | 1258 |  | <0.001 |
|  | Women | 65-69 | 517 | 458 | 374 | 365 | 259 | 241 | <0.001 | <0.001 |
|  |  | 70-74 | 1040 | 779 | 680 | 598 | 500 | 413 |  | 0.001 |
|  |  | 75-79 | 1814 | 1478 | 1182 | 1019 | 841 | 685 |  | <0.001 |
|  |  | 80-84 | 3025 | 2368 | 2007 | 1689 | 1356 | 1114 |  | <0.001 |
| Joint disorders | Men | 65-69 | 361 | 314 | 286 | 272 | 242 | 238 | <0.001 | 0.001 |
|  |  | 70-74 | 625 | 552 | 479 | 489 | 424 | 453 |  | 0.01 |
|  |  | 75-79 | 818 | 733 | 635 | 673 | 631 | 573 |  | 0.009 |
|  |  | 80-84 | 849 | 731 | 734 | 726 | 702 | 722 |  | 0.09 |
|  | Women | 65-69 | 833 | 776 | 747 | 653 | 588 | 666 | <0.001 | 0.02 |
|  |  | 70-74 | 1328 | 1228 | 1198 | 1226 | 1093 | 1114 |  | 0.01 |
|  |  | 75-79 | 1469 | 1378 | 1340 | 1446 | 1332 | 1427 |  | 0.67 |
|  |  | 80-84 | 1390 | 1288 | 1254 | 1358 | 1181 | 1342 |  | 0.54 |
| Fractures | Men | 65-69 | 163 | 163 | 129 | 113 | 121 | 102 | <0.001 | 0.008 |
|  |  | 70-74 | 211 | 197 | 160 | 167 | 153 | 139 |  | 0.004 |
|  |  | 75-79 | 256 | 252 | 196 | 225 | 216 | 215 |  | 0.16 |
|  |  | 80-84 | 338 | 328 | 310 | 302 | 297 | 320 |  | 0.16 |
|  | Women | 65-69 | 248 | 244 | 217 | 228 | 212 | 211 | 0.58 |  |
|  |  | 70-74 | 360 | 363 | 332 | 336 | 355 | 315 |  |  |
|  |  | 75-79 | 495 | 519 | 452 | 467 | 499 | 516 |  |  |
|  |  | 80-84 | 693 | 745 | 679 | 693 | 748 | 738 |  |  |
| Osteoporosis | Men | 65-69 | 20 | 13 | 7 | 10 | 7 | 7 | <0.001 | 0.04 |
|  |  | 70-74 | 60 | 42 | 31 | 19 | 25 | 14 |  | 0.008 |
|  |  | 75-79 | 111 | 74 | 52 | 44 | 30 | 19 |  | 0.002 |
|  |  | 80-84 | 200 | 87 | 78 | 59 | 54 | 52 |  | 0.049 |
|  | Women | 65-69 | 300 | 225 | 184 | 164 | 138 | 120 | <0.001 | 0.002 |
|  |  | 70-74 | 625 | 439 | 404 | 337 | 285 | 274 |  | 0.006 |
|  |  | 75-79 | 837 | 566 | 510 | 494 | 443 | 357 |  | 0.01 |
|  |  | 80-84 | 832 | 582 | 534 | 521 | 424 | 384 |  | 0.009 |
| Ischemic heart disease | Men | 65-69 | 372 | 301 | 229 | 203 | 176 | 176 | <0.001 | 0.005 |
|  |  | 70-74 | 523 | 438 | 329 | 303 | 270 | 214 |  | 0.001 |
|  |  | 75-79 | 749 | 534 | 445 | 385 | 347 | 307 |  | 0.006 |
|  |  | 80-84 | 742 | 639 | 495 | 476 | 423 | 378 |  | 0.002 |
|  | Women | 65-69 | 287 | 219 | 145 | 123 | 91 | 72 | <0.001 | 0.002 |
|  |  | 70-74 | 447 | 359 | 241 | 190 | 155 | 126 |  | 0.002 |
|  |  | 75-79 | 585 | 494 | 339 | 272 | 221 | 166 |  | <0.001 |
|  |  | 80-84 | 727 | 598 | 463 | 386 | 309 | 232 |  | <0.001 |
| Diabetes mellitus | Men | 65-69 | 652 | 610 | 572 | 557 | 472 | 502 | <0.001 | 0.004 |
|  |  | 70-74 | 714 | 669 | 639 | 673 | 619 | 605 |  | 0.02 |
|  |  | 75-79 | 719 | 612 | 593 | 652 | 602 | 663 |  | 0.58 |
|  |  | 80-84 | 698 | 634 | 595 | 540 | 495 | 624 |  | 0.18 |
|  | Women | 65-69 | 525 | 473 | 426 | 383 | 327 | 344 | <0.001 | 0.002 |
|  |  | 70-74 | 710 | 575 | 534 | 543 | 447 | 440 |  | 0.007 |
|  |  | 75-79 | 834 | 664 | 579 | 593 | 498 | 490 |  | 0.009 |
|  |  | 80-84 | 750 | 701 | 624 | 604 | 489 | 545 |  | 0.007 |
| Hypertension | Men | 65-69 | 1522 | 1271 | 1082 | 1099 | 961 | 1045 | <0.001 | 0.02 |
|  |  | 70-74 | 1957 | 1791 | 1444 | 1421 | 1293 | 1337 |  | 0.01 |
|  |  | 75-79 | 2410 | 1905 | 1599 | 1704 | 1532 | 1668 |  | 0.07 |
|  |  | 80-84 | 2596 | 2011 | 1709 | 1872 | 1614 | 1780 |  | 0.07 |
|  | Women | 65-69 | 1984 | 1675 | 1395 | 1347 | 1105 | 1191 | <0.001 | 0.006 |
|  |  | 70-74 | 2782 | 2307 | 1949 | 1959 | 1570 | 1601 |  | 0.004 |
|  |  | 75-79 | 3325 | 2720 | 2306 | 2396 | 1995 | 2113 |  | 0.02 |
|  |  | 80-84 | 3537 | 2842 | 2614 | 2525 | 2266 | 2407 |  | 0.02 |
| Pneumonia | Men | 65-69 | 45 | 42 | 40 | 41 | 39 | 38 | 0.54 |  |
|  |  | 70-74 | 76 | 85 | 75 | 88 | 81 | 71 |  |  |
|  |  | 75-79 | 158 | 148 | 151 | 162 | 150 | 132 |  |  |
|  |  | 80-84 | 251 | 267 | 265 | 313 | 295 | 275 |  |  |
|  | Women | 65-69 | 25 | 18 | 18 | 21 | 19 | 16 | 0.75 |  |
|  |  | 70-74 | 38 | 38 | 38 | 31 | 32 | 28 |  |  |
|  |  | 75-79 | 61 | 66 | 65 | 66 | 63 | 55 |  |  |
|  |  | 80-84 | 112 | 136 | 118 | 133 | 143 | 126 |  |  |
| Malignant neoplasms | Men | 65-69 | 859 | 762 | 676 | 677 | 677 | 659 | <0.001 | 0.03 |
|  |  | 70-74 | 1127 | 1044 | 1009 | 1046 | 951 | 896 |  | 0.008 |
|  |  | 75-79 | 1265 | 1194 | 1160 | 1247 | 1199 | 1127 |  | 0.20 |
|  |  | 80-84 | 1301 | 1227 | 1127 | 1209 | 1199 | 1231 |  | 0.52 |
|  | Women | 65-69 | 442 | 419 | 389 | 379 | 397 | 391 | <0.001 | 0.08 |
|  |  | 70-74 | 561 | 518 | 486 | 501 | 457 | 472 |  | 0.02 |
|  |  | 75-79 | 650 | 590 | 522 | 533 | 549 | 520 |  | 0.055 |
|  |  | 80-84 | 650 | 577 | 522 | 514 | 512 | 533 |  | 0.07 |

The treatment rate is calculated as the estimated number of patients divided by the estimated population x 100,000.
