# Supplementary material for: The State of Health in Older Adults in Japan: Trends in Disability, Chronic Medical Conditions and Mortality
Source: PLoS One. 2015 Oct 2;10(10):e0139639. doi: 10.1371/journal.pone.0139639 (PMC4592221; doi:10.1371/journal.pone.0139639)
Supplement: S4 Table — The mortality rate is calculated as the number of deceased divided by the estimated population x 100,000. (DOCX) [file pone.0139639.s005.docx]

S4 Table. Trends in total mortality rate and mortality rates from specific causes in men and women from 1995 to 2010.

|  |  |  | Year | | | | p for overall trend | p for trend |
| --- | --- | --- | --- | --- | --- | --- | --- | --- |
|  |  |  | 1995 | 2000 | 2005 | 2010 |  |  |
| Total mortality | Men | 65-69 | 2002.8 | 1818.3 | 1559.7 | 1460.9 | <0.001 | 0.01 |
|  |  | 70-74 | 3154.7 | 2865.5 | 2637.3 | 2270.9 |  | 0.004 |
|  |  | 75-79 | 5461.1 | 4561.5 | 4401.7 | 3959.4 |  | 0.04 |
|  |  | 80-84 | 9484.5 | 8052.4 | 7328.5 | 7046.3 |  | 0.048 |
|  | Women | 65-69 | 864.2 | 751.5 | 659 | 596.3 | 0.003 | 0.008 |
|  |  | 70-74 | 1513.4 | 1244.5 | 1130.4 | 980.2 |  | 0.02 |
|  |  | 75-79 | 2814.8 | 2265.4 | 1998 | 1787.9 |  | 0.03 |
|  |  | 80-84 | 5429.7 | 4334.5 | 3870.6 | 3434.2 |  | 0.03 |
| Cerebrovascular diseases | Men | 65-69 | 226.5 | 176.8 | 141.4 | 115.3 | 0.004 | 0.01 |
|  |  | 70-74 | 418.1 | 321.9 | 264.5 | 199.6 |  | 0.007 |
|  |  | 75-79 | 851.8 | 605 | 500.6 | 388.5 |  | 0.02 |
|  |  | 80-84 | 1707.4 | 1226.9 | 941.3 | 748.3 |  | 0.02 |
|  | Women | 65-69 | 114.4 | 85.5 | 66 | 48.4 | 0.006 | 0.007 |
|  |  | 70-74 | 240.3 | 168.1 | 127.6 | 87.5 |  | 0.01 |
|  |  | 75-79 | 533.9 | 358.7 | 264.8 | 187.8 |  | 0.02 |
|  |  | 80-84 | 1175.9 | 800 | 592.4 | 413.5 |  | 0.02 |
| Heart diseases | Men | 65-69 | 254.5 | 227.6 | 212.2 | 190 | 0.003 | 0.005 |
|  |  | 70-74 | 432 | 371.6 | 351.5 | 291.1 |  | 0.02 |
|  |  | 75-79 | 801 | 630.1 | 607.5 | 523.1 |  | 0.05 |
|  |  | 80-84 | 1488.2 | 1173.9 | 1078.1 | 993.8 |  | 0.057 |
|  | Women | 65-69 | 113.2 | 93.7 | 83 | 64.2 | 0.006 | 0.006 |
|  |  | 70-74 | 237.6 | 181.7 | 164.8 | 128.6 |  | 0.02 |
|  |  | 75-79 | 507.8 | 398.9 | 343 | 292 |  | 0.02 |
|  |  | 80-84 | 1052.1 | 844.4 | 749.7 | 639.9 |  | 0.02 |
| pneumonia | Men | 65-69 | 90.8 | 79.1 | 69.6 | 62.8 | 0.006 | 0.007 |
|  |  | 70-74 | 207.8 | 191.4 | 172 | 147.6 |  | 0.004 |
|  |  | 75-79 | 526.9 | 435.6 | 428.2 | 364.8 |  | 0.04 |
|  |  |  | 1225.8 | 1084.1 | 985.8 | 906.4 |  | 0.009 |
|  | Women | 65-69 | 32.6 | 27.3 | 22.4 | 18.5 | 0.01 | 0.002 |
|  |  | 70-74 | 83.3 | 63.8 | 56.5 | 47 |  | 0.03 |
|  |  | 75-79 | 216.5 | 168.3 | 142 | 118.5 |  | 0.02 |
|  |  | 80-84 | 566.9 | 445.4 | 384.1 | 323.6 |  | 0.02 |
| Malignant neoplasms | Men | 65-69 | 921.6 | 869.5 | 720.8 | 684.9 | <0.001 | 0.03 |
|  |  | 70-74 | 1271.1 | 1255.6 | 1160.7 | 1002.5 |  | 0.06 |
|  |  | 75-79 | 1766.3 | 1685.3 | 1672.9 | 1539.3 |  | 0.05 |
|  |  | 80-84 | 2359.8 | 2312.4 | 2230.8 | 2225.9 |  | 0.04 |
|  | Women | 65-69 | 358.5 | 340.2 | 305 | 296.4 | <0.001 | 0.02 |
|  |  | 70-74 | 513.4 | 482.9 | 456.2 | 416.1 |  | 0.004 |
|  |  | 75-79 | 736.9 | 691.7 | 648.9 | 614.5 |  | 0.002 |
|  |  | 80-84 | 1054.4 | 992.9 | 954.6 | 900.4 |  | 0.004 |

The mortality rate is calculated as the number of deceased divided by the estimated population x 100,000.
